# The Initial Step in Human Immunodeficiency Virus Type 1 GagProPol Processing Can Be Regulated by Reversible Oxidation

**DOI:** 10.1371/journal.pone.0013595

**Published:** 2010-10-22

**Authors:** Sarah I. Daniels, David A. Davis, Erin E. Soule, Stephen J. Stahl, Irene R. Tebbs, Paul Wingfield, Robert Yarchoan

**Affiliations:** 1 HIV and AIDS Malignancy Branch, Center for Cancer Research, National Cancer Institute, National Institutes of Health, Bethesda, Maryland, United States of America; 2 Protein Expression Laboratory, National Institute of Arthritis and Musculoskeletal and Skin Diseases, National Institutes of Health, Bethesda, Maryland, United States of America; University of California San Francisco, United States of America

## Abstract

**Background:**

Maturation of human immunodeficiency virus type 1 (HIV-1) occurs upon activation of HIV-1 protease embedded within GagProPol precursors and cleavage of Gag and GagProPol polyproteins. Although reversible oxidation can regulate mature protease activity as well as retrovirus maturation, it is possible that the effects of oxidation on viral maturation are mediated in whole, or part, through effects on the initial intramolecular cleavage event of GagProPol. In order assess the effect of reversible oxidation on this event, we developed a system to isolate the first step in protease activation involving GagProPol.

**Methodology/Principal Findings:**

To determine if oxidation influences this step, we created a GagProPol plasmid construct (pGPfs-1C) that encoded mutations at all cleavage sites except p2/NC, the initial cleavage site in GagProPol. pGPfs-1C was used in an *in vitro* translation assay to observe the behavior of this initial step without interference from subsequent processing events. Diamide, a sulfhydral oxidizing agent, inhibited processing at p2/NC by >60% for pGPfs-1C and was readily reversed with the reductant, dithiothreitol. The ability to regulate processing by reversible oxidation was lost when the cysteines of the embedded protease were mutated to alanine. Unlike mature protease, which requires only oxidation of cys95 for inhibition, both cysteines of the embedded protease contributed to this inhibition.

**Conclusions/Significance:**

We developed a system that can be used to study the first step in the cascade of HIV-1 GagProPol processing and show that reversible oxidation of cysteines of HIV-1 protease embedded in GagProPol can block this initial GagProPol autoprocessing. This type of regulation may be broadly applied to the majority of retroviruses.

## Introduction

HIV-1 GagProPol autoprocessing is a unique step in the HIV life cycle and involves the dimerization of two GagProPol monomers and subsequent autocleavage of the polyproteins to generate virus structural proteins as well as protease, reverse transcriptase, and integrase [1]. This autoprocessing is usually delayed until GagProPol and Gag polyproteins assemble at the cell membrane and is followed by further cleavage of Gag and other GagProPol polyproteins during and/or just after budding from the plasma membrane [2], [3]. Premature autoprocessing and activation of protease in HIV-1 infected cells or over-expression of GagProPol, impairs viral production and infectivity [4], [5]. Similar results have been described for Rous sarcoma virus (RSV), where a protease-linked dimer results in premature viral processing in the cytoplasm of infected cells in conjunction with decreased virus production [6]. These studies indicate a need for control over the timing of protease activation, particularly when viral expression and therefore protease are at their highest levels.

Previous studies have demonstrated that reversible oxidation can regulate maturation of immature HIV-1 virions [7]. One possible way that this may occur is through modification of the mature protease by oxidation of a cysteine at position 95 (cys95) at the dimer interface; this could then interfere with protease dimerization. In the case of HIV-2, reversible oxidation of methionine 95 (met95) serves to regulate protease activity in the same way [8], [9]. For HTLV-1, two conserved cysteines at positions 90 and 109 serve a similar function [8]. The oxidative modifications of HIV-1 and HIV-2 protease interfere with dimerization [8], [9] and it is predicted that the modifications of HTLV-1 protease may prevent dimerization as well.

A similar process is involved in the regulation of the maturation of Mason-Pfizer monkey virus (M-PMV) capsids [10]. M-PMV, also a retrovirus, is unusual in that the GagPro polyproteins assemble in the cytoplasm but the embedded protease, which contains two methionines and one cysteine at the dimer interface, is still not activated until the time of budding. Experimentally, the M-PMV protease and viral maturation can be activated by the addition of a reducing agent [10]. In fact, essentially all retroviral proteases studied have one or more oxidizable residues at or near the protease dimer interface with the potential to be regulated in this manner [8]. Cellular enzymes, such as thioltransferase and methionine sulfoxide reductase have been shown to reverse these cysteine and methionine oxidative modifications, respectively, leading to protease dimerization and reactivation [8], [11], [12]. It has been suggested that that regulation by reversible oxidation of one or more cysteine or methionine residues at or near the dimer interface is a general property of retroviral proteases [8].

However, it remains unclear whether the regulation of viral maturation by reversible oxidation is effected primarily through oxidation of mature protease or whether the main target is the initial autoprocessing event by the GagProPol polyprotein. The first step in HIV-1 polyprotein processing is an intramolecular cleavage between the p2 protein (also known as spacer peptide 1 or SP1) domain and nucleocapsid (NC) domain or p2/NC [13]–[15]. This step is unique from those carried out by mature protease in that: 1) cleavage of GagProPol in trans by exogenous protease at the p2/NC junction is much more rapid than cleavage at p2/NC by the dimerized precursor polyprotein [13]; 2) the initial processing step is quite resistant to inhibition by active site protease inhibitors when compared to mature protease [14 and our unpublished data]; and 3) even the second cleavage carried out by the embedded protease is 27 fold more sensitive to the potent active site inhibitor, ritonavir, as compared to the initial cleavage at p2/NC by the embedded protease. Due to these differences it was unclear whether oxidation of the embedded protease in GagProPol could reversibly inhibit the initial step in viral maturation.

In this report, we developed a unique assay that enabled us to isolate the initial GagProPol autoprocessing event without interference from subsequent proteolytic events. Through modification of a GagProPol encoding plasmid, we were able to measure the initial cleavage of GagProPol at the P2/NC site. This allowed us to assess conditions that may control this cleavage event. We demonstrate that the initial step in GagProPol processing can be specifically and reversibly regulated through oxidation of the cysteines of the embedded HIV-1 protease, and that this regulation differs in some ways from that of mature HIV-1 protease. The observations reported here indicate reversible oxidation as a potential mechanism to regulate the onset of retroviral polyprotein processing.

## Results

### Development of a GagProPol first-cut assay

**Figure 1:**
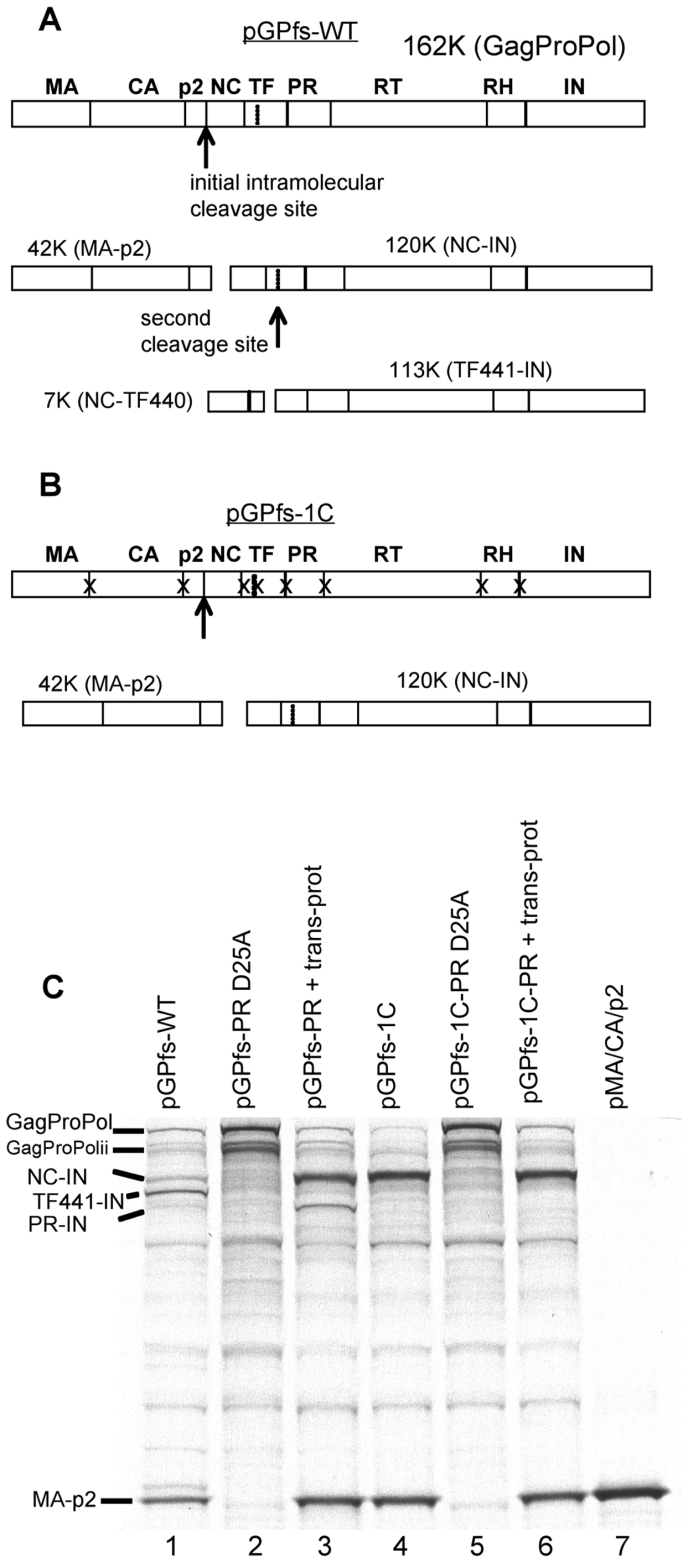
Schematic diagram for the GagProPol polyprotein precursor generated from pGPfs, the polyprotein precursor generated from pGPfs-1C and results of GagProPol proteolytic processing of pGPfs (WT) and pGPfs-1C. A) The GagProPol plasmid construct, pGPfs, encodes a 162 kDa polyprotein. The processing sites for the polyprotein are indicated with vertical lines. The location of the first cut site (p2/NC) is indicated with an arrow. The known secondary cut site (occurring between amino acids 440 and 441 within the transframe protein) is indicated by a dotted line. The resultant products from cleavages at these two sites are indicated. B) The schematic for pGPfs-1C (modified pGPfs plasmid) showing the sites for the blocking mutations designated with an X. C) *In vitro* transcription/translation with ^35^S-methionine was carried out for 1 h in the presence of the indicated plasmid construct. Note that plasmids pGPfs-PR D25A and pGPfs-1C-PR D25A encode a protease with a D25A mutation rendering it inactive. pMA/CA/p2, yields the small product (MA-p2) fragment and was used to verify the MA-p2 fragment. Exogenous HIV-1 protease provided in trans (trans-prot, 250 nM) was added to pGPfs-PR D25A or pGPfs-1C-PR D25A constructs following 45 min of translation and was terminated 15 minutes later with 3X LDS sample buffer. Samples were separated by LDS-PAGE and visualized by autoradiography. The location for GagProPol, the internal initiation GagProPol (GagProPol ii) and the products of GagProPol processing (MA-p2, NC-IN, TF441-IN, and PR-IN) occurring through the action of the embedded protease or the exogenous protease are indicated in the figure.

The pGPfs, developed by Pettit et al. [13], contains a frame-shift mutation that causes it to produce full length GagProPol but not p55 Gag protein. The GagProPol produced by this plasmid subsequently undergoes limited autoproteolytic processing. The major products resulting from GagProPol autoprocessing in this system are a result of cleavage at p2/NC and cleavage within the transframe protein (TF) yielding MA-p2, NC-IN, NC-TF440 and TF441-IN (Figure 1A) [13]. We were interested in studying the initial step of polyprotein processing and in determining if this step could be regulated by reversible oxidation. To this end, we produced a modified construct that would only undergo the initial step of polyprotein processing. To ensure processing at only the p2/NC site, we incorporated blocking mutations at all other major processing sites within pGPfs. Amino acid mutations flanking the GagProPol processing sites were chosen based upon previous studies indicating that these substitutions prevented proteolytic processing (Table 1) [16]-[18]. In addition, alanine mutations were placed at the processing sites flanking the N and C termini of protease to prevent processing at these sites by the embedded protease [13], while the protease sequence itself was conserved as wild type (Table 1). While cleavage at these flanking sites was not previously detected [13], these mutations provided additional assurance that the results would not be skewed by a small amount of this product. These modifications also reduced the likelihood that partially processed GagProPol could cleave other polyproteins in *trans*. The modified pGPfs plasmid had eight processing site mutations and was designated as pGPfs-one cut (pGPfs-1C) (Figure 1B).

**Table 1:** Processing site and protease mutations incorporated into pGPfs-1C.

Processing site	Nucleotide sequence	Amino acid sequence
p17/p24 WT	CAA AAT TAC/CCT ATA GTG	Gln Asn Tyr/Pro Ile Val
p17/p24 Mut	CAA AAT **AT**C/C**T**T ATA GTG	Gln Asn **Ile**/**Leu** Ile Val
p24/p2 WT	AGA GTT TTG/GCT GAA GCA	Arg Val Leu/Ala Glu Ala
p24/p2 Mut	AGA GTT **G**TG/GCT GAA GCA	Arg Val **Val**/Ala Glu Ala
NC/TF WT	CAG GCT AAC/TTC CTC CGC	Gln Ala Asn/Phe Leu Arg
NC/TF Mut	CAG GCT A**T**C/**GAT** CTC CGC	Gln Ala **Ile/Asp** Leu Arg
TF440/441 WT	TTG GCC TTC/CTA CAA GGG	Leu Ala Phe/Leu Gln Gly
TF440/441 Mut	TTG GCC **A**TC/CTA CAA GGG	Leu Ala **Ile**/Leu Gln Gly
TF/Protease WT	TTT AAC TTC/CCT CAG GTC	Phe Asn Phe/Pro Gln Val
TF/Protease Mut	**GCG GC**C **GC**C/CCT CAG GTC	**Ala Ala Ala**/Pro Gln Val
Protease/Pol WT	TTA AAT TTT/CCC ATT AGC	Leu Asn Phe/Pro Ile Ser
Protease/Pol Mut	TTA AAT TTT/**G**C**TGCAGC**C	Leu Asn Phe/**Ala** **Ala Ala**
Pol/RNaseH WT	GAA ACC TTC/TAT GTA GAT	Glu Thr Phe/Tyr Val Asp
Pol/RNaseH Mut	GAA **A**CC AT**C**/TAC GTA GAT	Glu Thr **Ile**/Tyr Val Asp
RNaseH/Integrase WT	AAA GTA CTA/TTT TTA GAT	Lys Val Leu/Phe Leu Asp
RNaseH/Integrase Mut	AAA GTA **A**TA/TTT TTA GAT	Lys Val **Ile**/Phe Leu Asp

Protease Mutations	Nucleotide sequence	Amino acid sequence
D25 WT	CTA TTA GAT ACA GGA	Leu Leu Asp Thr Gly
A25 Mut	CTA TT**G** **GCC** ACA GGA	Leu Leu **Ala** Thr Gly
C67 WT	GAA ATC TGT GGA CAT	Glu Ile Cys Gly His
A67 Mut	GAA ATC **GCC** GGA CAT	Glu Ile **Ala** Gly His
C95 WT	ATT GGT TGC ACT TTA	Ile Gly Cys Thr Leu
A95 Mut	ATT GGT **GCA** ACT TTA	Ile Gly **Ala** Thr Leu

*In vitro* translation was carried out using wild type (WT) pGPfs or pGPfs-1C. As expected, *in vitro* translation of the WT plasmid generated GagProPol, truncated GagProPol from internal initiations (GagProPolii) and expected products of the first and second processing steps (MA-p2, NC-IN, TF441-IN, Figure 1C) [13], [14], [19]. These products (MA-p2, NC-IN, and TF441-IN) were absent when a pGPfs containing a D25A mutation in the protease gene that inactivates the embedded protease (pGPfs-PR D25A) was utilized (Figure 1C, lane 2). A number of weaker non-specific bands were also produced by the WT pGPfs, similar to those seen previously [13], [14], [19]. Most of these weaker bands are unrelated to GagProPol processing as they are generated both when using GagProPol plasmids encoding inactive as well as active protease (Figure 1C, lane 2 and 5). Addition of purified HIV-1 protease (15 min incubation) to pGPfs-PR D25A yielded the major products MA-p2, NC-IN and a band tentatively identified as PR-IN based on studies reported previously (Figure 1C, lane 3) [13].

In contrast to the results with WT pGPfs, *in vitro* translation of pGPfs-1C yielded only the major products of the first processing step and these aligned with the previously identified products obtained from WT pGPfs, namely MA-p2 and NC-IN (Figure 1C, lane 4). In particular, pGPfs-1C did not generate any of the additional processing intermediates that were seen with pGPfs. To confirm that the products from pGPfs-1C processing were indeed due to processing by the embedded protease, we created a D25A mutation in the protease region of pGPfs-1C and designated the plasmid containing inactive protease as pGPfs-1C-PR D25A. *In vitro* translation of pGPfs-1C-PR D25A produced full length GagProPol (and GagProPolii) but did not produce any cleavage products (Figure 1C, lane 5). As expected, addition of purified HIV-1 protease for 15 minutes (250 nM dimer) to the sample producing D25A GagProPol resulted in almost complete processing only at the p2/NC cut site (Figure 1C, lane 6). To further verify that processing was occurring at the p2/NC cut site, we generated a plasmid encoding MA/CA/p2 and used it as a standard in the *in vitro* translation assay. The protein product generated from this plasmid (MA-p2) migrated to the same position on the gel as the small product generated from the GagProPol encoded by the first cut plasmid (Figure 1C, lane 7). These studies indicated that *in vitro* translation of pGPfs-1C produced GagProPol that undergoes a single cleavage event at p2/NC.

**Figure 2:**
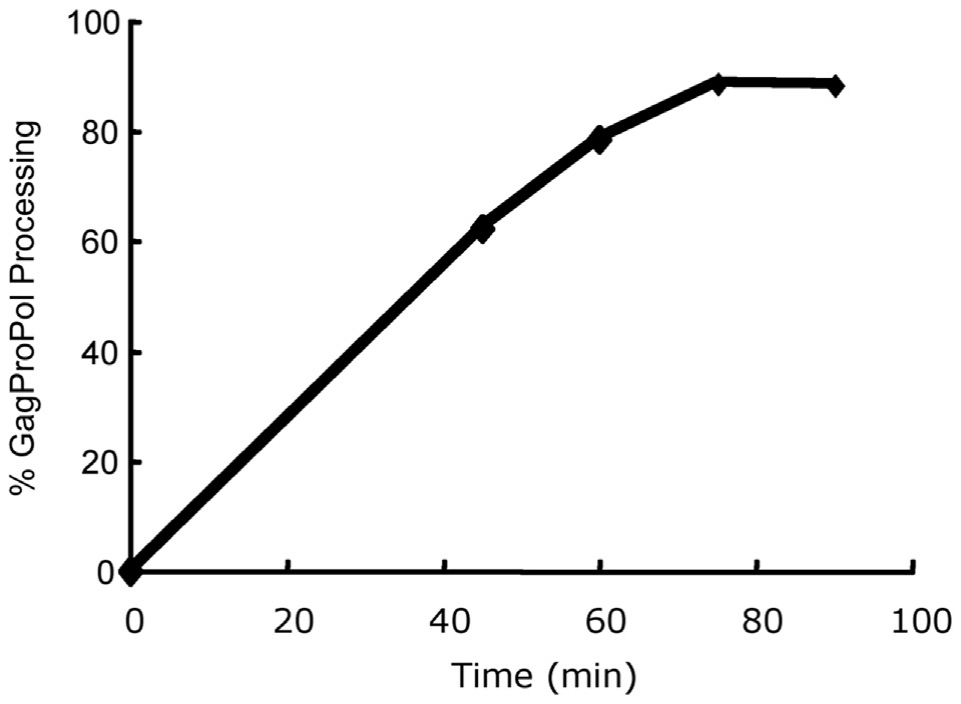
Time course for pGPfs-1C GagProPol processing. *In vitro* transcription translation with ^35^S-methionine was carried out for 45, 60, 75 and 90 minutes. Samples were separated by LDS-PAGE and visualized by autoradiography and the percent processing determined using densitometry. The precursors (GagProPol and GagProPolii) and two products (MA-p2, NC-IN) were scanned and the extent of processing calculated as a percent: [(products)/(precursor plus products) x 100]. The LDS-PAGE for this data is shown Figure S1.

The rate of processing at the initial cleavage site was investigated in a time course experiment. Approximately 60% of the pGPfs-1C GagProPol underwent cleavage at the p2/NC within 45 minutes and 90% underwent cleavage within 75 minutes, at which time it reached a plateau (Figure 2 and Figure S1). After two hours of incubation, minor bands below the large product were evident but these represented less than 5% of the total products produced (data not shown). These likely represent alternate, albeit much less favorable, cleavages occurring through the action of the embedded protease within the large product (NC-IN). These data indicate the embedded HIV-1 protease readily cleaves within pGPfs-1C GagProPol at the p2/NC junction yielding MA-p2 and NC-IN products and that the blocking mutations do not adversely affect processing at the initial site. However, the presence of blocking mutations prevents any significant processing of the polyprotein at the other cleavage sites.

### Inhibition of the initial step in GagProPol Processing by oxidation is dependent on the cysteines of HIV-1 protease

**Figure 3:**
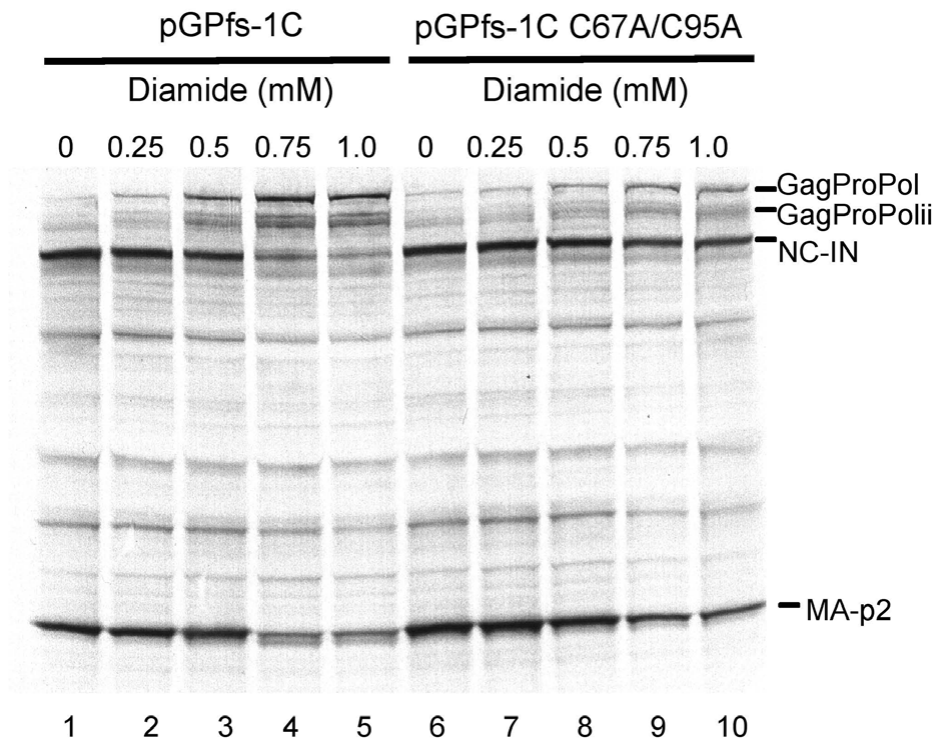
Dose dependent inhibition of pGPfs-1C GagProPol and pGPfs-1C C67A/C95A GagProPol processing by diamide. *In vitro* transcription/translation with ^35^S-methionine was carried out in the presence of increasing concentrations of the sulfhydral oxidant diamide (A) pGPfs-1C (lanes 1–5) or the double cysteine mutant construct pGPfs-1C C67A/C95A (lanes 6–10). Samples were separated by LDS-PAGE and visualized by autoradiography. The precursors (GagProPol and GagProPolii) and products (MA-p2, NC-IN) of GagProPol processing by the embedded protease are indicated.

We previously demonstrated that HIV-1 protease could be regulated by reversible oxidation of the conserved cysteines of HIV-1 protease and found that exposure of immature HIV-1 virions to oxidizing agents could inhibit viral maturation [7], [11], [20]. This inhibition required the presence of cys95, but not cys67, since replacing cys95 with alanine led to processing even in the presence of oxidizing agents, while replacing cys67 with alanine had no substantial effect [20]. However, it was unclear if oxidation could similarly affect the action of the embedded protease at the initial step of GagProPol processing. To determine if the initial step in GagProPol processing was sensitive to oxidation, we examined the effect of oxidants on GagProPol processing using pGPfs-1C. Initial studies revealed that hydrogen peroxide (1–10 mM), a nonspecific oxidizing agent, caused partial inhibition of GagProPol processing in a cysteine-dependent manner (data not shown). However, higher concentrations of hydrogen peroxide were detrimental to the translation system, thereby preventing any further assessment on GagProPol processing. Diamide is a thiol-oxidizing agent that can create mixed disulfide bonds between proteins and low molecular weight thiols and therefore we tested the effect of diamide on GagProPol processing [21], [22]. In the absence of diamide, processing with pGPfs-1C proceeded almost to completion as evidenced by the presence of the two GagProPol processing products and the absence of full length GagProPol (Figure 3, lane 1). However, in the presence of diamide, GagProPol processing was inhibited in a dose-dependent fashion at concentrations that only had a minor effect on the *in vitro* translation system as evidenced by the total product produced in the reactions (Figure 3, lanes 2–5). By contrast, the effect of diamide on the processing of pGPfs-1C C67A, C95A GagProPol was much less pronounced (Figure 3, lanes 6–10). These data indicate that the cysteines of HIV-1 protease are particularly susceptible to diamide oxidation and this oxidation leads to inhibition of the initial cleavage event.

**Figure 4:**
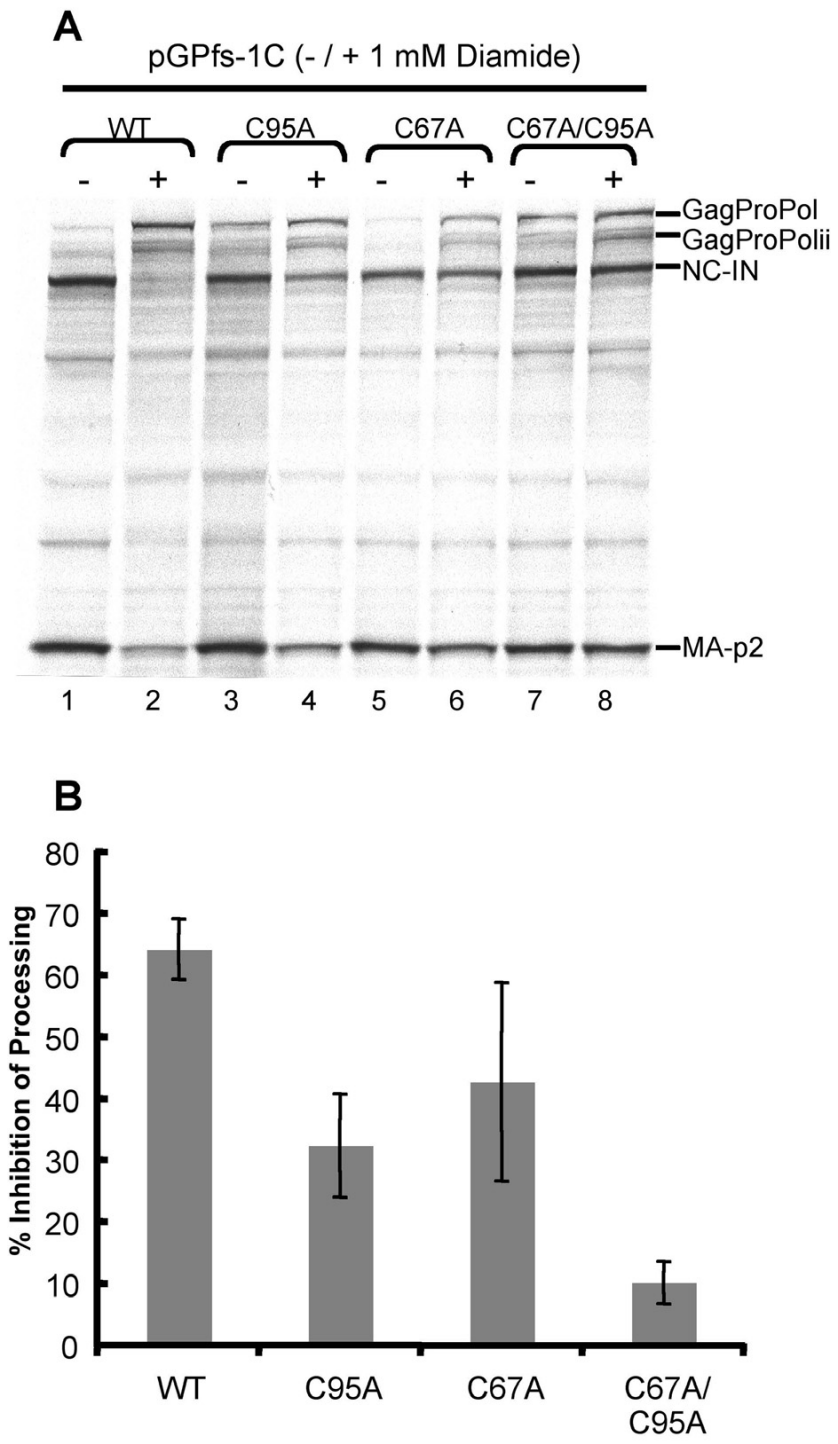
Inhibition of GagProPol processing by the oxidant diamide is dependent on both cysteines of the embedded HIV-1 protease. (A) *In vitro* transcription/translation with ^35^S-methionine was carried out for 1 h in the absence (−) or presence (+) of 1 mM diamide. Samples were separated by LDS-PAGE and visualized by autoradiography. The precursors and products of GagProPol processing by the embedded protease are indicated. The pGPfs-1C plasmid construct used is indicated above the wells. (B) Densitometry was carried out on autoradiograms from 4 separate experiments under the conditions as described for Figure 4A and the extent of inhibition of processing calculated. The percent inhibition was calculated as (processing without diamide-processing with diamide)/(processing without diamide) x 100. Percent processing is calculated as in Figure 2. Values represent the average +/− the standard deviation from 4 independent experiments.

To assess the contribution of each cysteine within the embedded protease to inhibition of processing by diamide, we examined GagProPol processing for the plasmid constructs pGPfs-1C, pGPfs-1C C95A, pGPfs-1C C67A and pGPfs-1C C67A C95A in the presence and absence of 1 mM diamide. In the absence of diamide, all four GagProPols underwent substantial processing, although there were some small differences among them. Of note, the plasmids retaining cys95 (pGPfs-1C and pGPfs-1C C67A) processed nearly to completion (Figure 4A, lanes 1 and 5) while the plasmids lacking cys95 (pGPfs-1C C95A pGPfs-1C C67A C95A) had some residual unprocessed GagProPol remaining (Figure 4A, lanes 3 and 7). This was a consistent finding throughout our studies and indicates that the presence of cys95 is important for maximally efficient cleavage of GagProPol at p2/NC. Diamide (1 mM) substantially inhibited processing of pGPfs-1C GagProPol, but was less effective on each of the single cysteine mutants (Figure 4A, lanes 1–6) and was the least effective at inhibiting processing of the double cysteine mutant GagProPol produced from pGPfs-1C C67A, C95A (Figure 4A, lanes 7–8). Thus, the loss of either cysteine significantly impaired the ability of diamide to block processing. To quantify this effect among the different plasmids and correct for internal differences in translation and processing of the alanine mutants, densitometry was performed on the unprocessed GagProPol bands and product bands from four separate experiments. The extent of processing was first calculated for the untreated controls and then compared to processing following treatment with 1 mM diamide. As shown in Figure 4B, diamide inhibited the processing of GagProPol produced from pGPfs-1C about 65% while processing for C95A, C67A and the double mutant was inhibited by 35%, 42%, and 10%, respectively (Figure 4B). These data demonstrate that optimal inhibition of GagProPol processing by diamide requires the presence of both cys67 and cys95.

### Oxidative inhibition of the initial step in GagProPol processing is reversible

**Figure 5:**
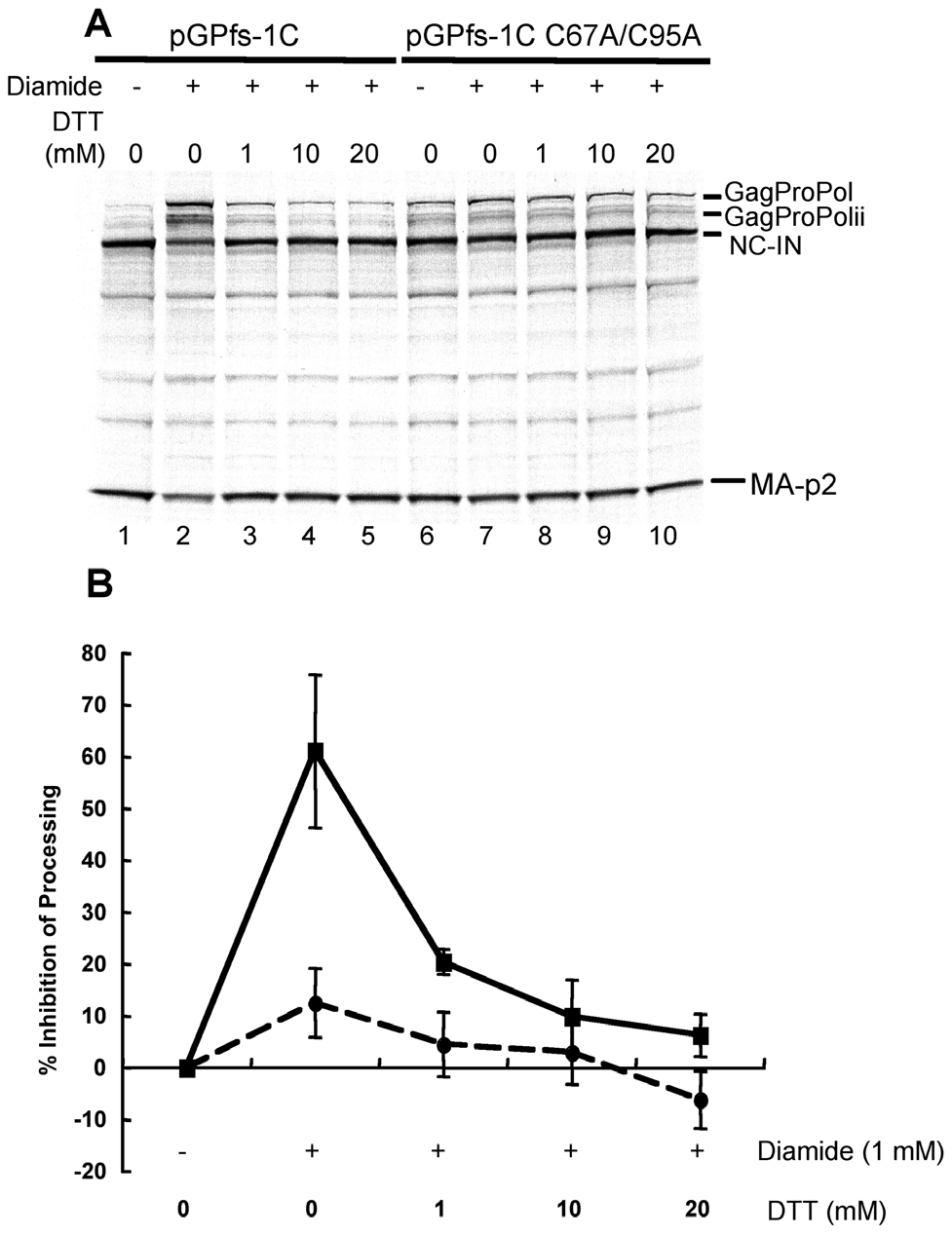
Dose response restoration of polyprotein processing by DTT following inhibition of pGPfs-1C or pGPfs-1C C67A,C95A processing by diamide. A) *In vitro* transcription/translation was carried out with pGPfs-1C (lanes 1–5) or pGPfs-1C C67A,C95A (lanes 6–10) in the absence (−) or presence (+) of 1 mM diamide for 45 min followed by addition 1, 10, or 20 mM DTT for 15 minutes as shown above each lane. Samples were separated by LDS-PAGE and analyzed by autoradiography. The precursors and products of GagProPol processing by the embedded protease are indicated. B) Densitometry was carried out on autoradiograms from 3 separate experiments under the conditions as described for Figure 5A. The percent inhibition of processing was calculated and plotted. for pGPfs-1C (ν) solid line and pGPfs-1C C67A,C95A (λ) dotted line. The following formula was used: Percent inhibition of processing  =  (processing without diamide-processing with diamide and 0–20 mM DTT)/(processing without diamide) x 100. The values represent the average +/− the standard deviation for 3 independent experiments.

Next, we wanted to assess the ability of DTT, a sulfhydral reducing agent, to restore processing after inhibition by diamide treatment. To determine if DTT could reverse the inhibition of GagProPol processing after an initial diamide treatment, the *in vitro* translation reaction was first carried out for 45 minutes in the absence or presence of 1 mM diamide. Following this, samples were treated with 0, 1, 10, or 20 mM DTT and the reaction was allowed to proceed for 15 more minutes. DTT reversed the diamide-induced inhibition of GagProPol processing at the first cut site (p2/NC) and did so in a dose dependent manner (Figure 5A, lanes 1–5). There was a low level of diamide induced inhibition of processing for the double cysteine mutant pGPfs-C C67A, C95A but this was restored following DTT treatment (Figure 5A, lanes 6–10). Densitometric analyses were done to assess the extent of reversal of diamide inhibited GagProPol processing for pGPfs-1C for three separate experiments. As shown in Figure 5B, diamide inhibited 60% of the processing and much of this (67%) was restored with as little as 1 mM DTT and almost 90% was restored with 20 mM DTT (Figure 5B). Overall, these data indicate that diamide oxidation of the cysteines within the embedded protease leads to inhibition of the initial cleavage step in GagProPol polyprotein processing and this inhibition can be readily reversed with reducing agent.

**Figure 6:**
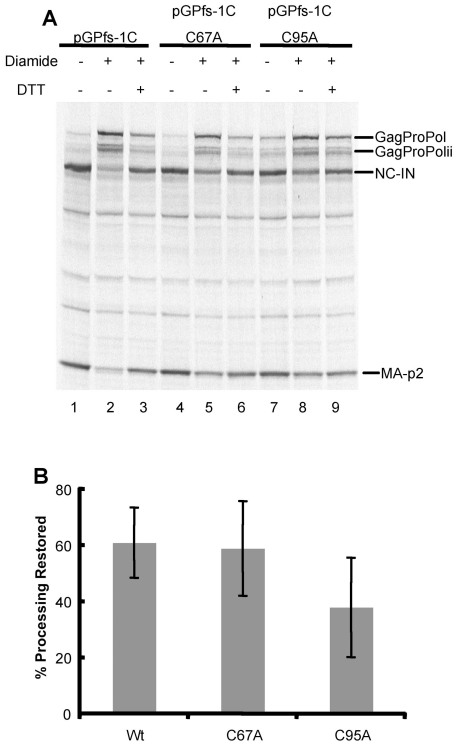
Restoration of polyprotein processing by DTT following inhibition of pGPfs-1C, pGPfs C95A, or pGPfs C67A processing by diamide. A) *In vitro* transcription/translation was carried out for pGPfs-1C, pGPfs C95A, or pGPfs C67A in the absence (−) or presence (+) of 1 mM diamide for 45 min followed by addition of 1 mM DTT. Samples were incubated for an additional 15 min to allow for processing to take place and then separated by LDS-PAGE and analyzed by autoradiography. The precursors and products of GagProPol processing by the embedded protease are indicated. B) Densitometry was carried out on autoradiograms from 3 separate experiments under the conditions as described for Figure 6A. The percent processing restored was calculated as (processing with DTT and diamide-processing with diamide)/(processing without DTT or diamide-processing with diamide) x 100. The values represent the average +/− the standard deviation for 3 independent experiments.

We also investigated the ability of DTT to reverse the diamide-induced inhibition of GagProPol processing for the single cysteine mutants. Once again, diamide blocked the majority of GagProPol processing obtained with pGPfs-1C, but was somewhat less effective at blocking GagProPol processing obtained with single cysteine mutant C67A or C95A pGPfs-1Cs (Figure 6A, lanes 2,5,9). Addition of 1 mM DTT, following inhibition by diamide, restored processing in all cases (Figure 6A, lanes 3,6,and 9). Although processing for pGPfs-1C C67A and pGPfs-1C C95A were similarly inhibited by diamide, restoration of processing with 1 mM DTT was most efficient with pGPfs-1C C67A, and similar to the reversal obtained for pGPfs-1C (Figure 5A). Based on densitometric analysis, DTT restored about 60% of the processing inhibited by diamide (Figure 6B). A similar improvement in GagProPol processing was seen with pGPfs-1C C67A that contains protease with cys95 intact (Figure 6B). However, DTT only restored an average of 40% of processing for the pGPfs-1C C95A GagProPol containing protease with cys67, although this lower level of processing was not statistically significant when compared to the other plasmids (Figure 6B). Together, these data suggest that oxidation of both cysteines can be reversed with DTT. However, cys67 may be more difficult to reverse when compared to cys95 of the embedded protease in GagProPol in the context of the single cysteine mutants.

## Discussion

Previous studies demonstrated that reversible oxidation inhibited HIV-1 maturation through the inhibition of protease activity and that the activity of mature protease could be regulated by such oxidation of residues at the dimer interface, thus interfering with dimerization[7], [8], [23]. However, the initial dimerization of HIV protease takes place between two GagProPol monomers that, after dimerization, undergo autoprocessing through the action of the embedded protease of the polyprotein. We hypothesized that regulation of this initial step by reversible oxidation of the dimer interface of GagProPol may account for much or all of the regulation of HIV viral maturation by redox changes. To explore this hypothesis, we used an *in vitro* GagProPol translation assay system to isolate the dimerization and first step in HIV-1 polyprotein processing. Our modified GagProPol plasmid construct yielded GagProPol that efficiently underwent dimerization and autocleavage at p2/NC without further processing taking place. Using this construct we found that autocleavage at p2/NC within GagProPol was quite sensitive to oxidation by the sulfhydral oxidant, diamide and this was readily reversible with reducing agent. The ability to regulate processing by reversible oxidation was lost when the cysteines of the embedded protease were mutated to alanine and processing proceeded irrespective to the addition of diamide. Although we could not determine the precise mechanism of inhibition using this assay, we suggest that the oxidative modification of cysteines in the embedded protease interferes with GagProPol dimerization, similar to that previously demonstrated for mature protease [8], [9]. Diamide oxidation also blocked the processing for pGPfs-1C C67A/C95A although this was less pronounced than that seen for the pGPfs-1C plasmid encoding a cysteine containing protease. The fact that the low level of inhibition with the double mutant was reversible with DTT (see Figure 5) suggests that other cysteines within the GagProPol also participate to some degree in the inhibition of processing. It has been reported that the nucleocapsid domain contains reactive cysteine residues which, when oxidized, reversibly affect polyprotein processing [24]. Therefore these residues may also play a partial role in the inhibition of GagProPol processing observed with diamide treatment.

There were some key differences between the effects of oxidation on the embedded protease and those observed for mature protease. For mature HIV-1 protease, oxidation of cys95 alone was sufficient to inactivate the enzyme and viral maturation [11], [20]. In contrast, the presence of both cysteines (67 and 95) was required to obtain maximum inhibition/regulation of the initial GagProPol processing step. Also, while oxidation of cys67 of mature protease by glutathionylation increased activity and stabilized the enzyme, oxidation of cys67 of the embedded protease within the *in vitro* translation system led to partial inhibition of GagProPol processing [11], [20]. This difference in the effect of cys67 oxidation of the embedded protease as compared to the mature protease is likely related to differences in the conformation of protease in GagProPol dimers compared to those for mature dimers. For mature protease, it is more difficult to reverse oxidation of cys67 versus that for cys95 [11]. While oxidation of cys95 in mature WT protease or C67A protease results in complete inactivation of the enzyme [11], [20] and inhibition of protease dimerization [8], [9], oxidation of GagProPol containing only cys95 leads only to partial inhibition of processing. Thus, as suggested previously [13], sequences within GagProPol that lie outside of the protease region may partially compensate for disruption of the protease dimeric interaction. We cannot rule out that the mutations that were made to prevent cleavage at subsequent processing sites had some influence on the differences observed between oxidation of endogenous and versus exogenous protease.

Reversible oxidation of certain amino acids of retroviral proteases has been suggested as one potential mechanism for preventing premature activation of the protease [8], [10]. Studies with immature HIV-1 virions produced by exposure to a protease inhibitor demonstrated that reducing agents could increase viral processing following removal of the inhibitor [7]. Similarly, immature capsids of M-PMV undergo proteolytic processing following the addition of reducing agent [10]. These studies implicated protease oxidation as a mechanism to inhibit protease activation. Our studies here demonstrate clearly that reversible oxidation can regulate the onset of the polyprotein processing cascade for HIV-1.

Evidence for the importance of redox-mediated regulation of protease and/or protease precursors is strongest in the case of M-PMV, in which the GagPro polyproteins assemble in the cytoplasm but do not mature until the time of budding [10]. However, similar regulation of retroviral protease has been shown for several other retroviruses, and essentially all retroviruses studied have at least one cysteine or methionine in the region of the dimer interface, suggesting that this regulation is constrained by viral evolution and therefore important in the retroviral lifecycle [7]–[9]. In the laboratory, HIV-1 does not require the presence of cys67 or cys95 to generate infectious virions from infected cells [7]. Nonetheless, cys67 and cys95 are highly conserved among HIV-1 isolates [25]. Therefore, for HIV-1, it is possible that the regulation of the initial step in processing by reversible oxidation may only be necessary in certain cell types or under certain conditions, such as those involving oxidative stress. Virus infection, including HIV-1 infection, leads to a state of oxidative stress in certain cell types and this is known to enhance HIV-1 replication through the activation of NF-κB [26]–[29]. Oxidative environments, like those encountered in infected cells [26], [30], could lead to reversible cysteine oxidation of the embedded protease and as a result prevent premature protease activation and premature cell death when GagProPol is accumulating to high levels in the cell. Thus, the presence of cysteines within protease may optimize viral production by preventing deleterious effects of the protease on the infected cells. This effect may be particularly important in cells that produce virus for extended periods of time such as monocytes/macrophages. By contrast, the D-type retroviruses, such as M-PMV, may utilize reversible oxidation as the primary means to control protease activity as evidenced by the lack of particle processing in the absence of reducing agent and the activation of such processing with reducing agent [10].

The initial GagProPol processing step provides an additional target for the development of inhibitors of viral replication, a step separate from the processes mediated by the mature protease. While intramolecular enzymatic steps are inherently more difficult to inhibit through a classical mode of competitive inhibition as compared to trans cleavage [14 and references therein], it may be possible to develop inhibitors of GagProPol dimerization that could prevent protease activation and autoprocessing. It is possible the observed diamide inhibition of GagProPol processing in this study results from the inhibition of GagProPol dimerization, although further studies will be necessary to determine this. Recent studies have provided evidence that darunavir and similar compounds have the ability to block protease dimerization within cells [31]. We are currently screening active site inhibitors in this assay to determine what structural properties might favor inhibition of the initial processing step and how these properties relate to their ability to block protease activity and protease dimerization. Moreover, compounds designed as dimerization inhibitors of protease can be screened to assess their potential as inhibitors of the initial processing step. It may be more feasible to inhibit the dimerization of GagProPol than to reverse the dimerization of the mature protease dimer considering the low dissociation constant of mature protease [32]. Such studies can provide an additional target for the pursuit of new therapies, especially in the treatment of multi-drug resistant HIV [33].

## Materials and Methods

### Plasmid construction and mutagenesis

The plasmids pGPfs and pGPfs-PR D25Awere obtained from Dr. Ronald Swanstrom (UNC-Chapel Hill, NC). pGPfs contains a T7 promoter and has a forced frame-shift mutation leading to the production of full length GagProPol in an *in vitro* translation system [13]. pGPfs-PR D25A contains a D25A mutation in the protease coding region to inactivate protease activity and prevent proteolytic processing of the GagProPol precursor generated during *in vitro* translation. Further mutations were introduced into pGPfs by site directed mutagenesis using a Stratagene QuikChange Multi Site-Directed Mutagenesis Kit. The primers used to introduce processing site and protease mutations are shown in Table 1. A DNA fragment encoding the matrix-capsid-p2 polyprotein (MA-p2) was produced using PCR to add NdeI and BamHI restriction sites [34]. This DNA fragment was then ligated into expression vector pET11 creating plasmid pMA/CA/p2 and its product was used as a control to verify the identity of the small product from the first processing step. Plasmids were also produced with single and double cysteine-to-alanine mutations and/or a D25A mutation introduced in the protease-coding region of pGPfs-1C. The primers used for these constructs are shown in Table 1.

### *In-vitro* translation reactions for studying GagProPol proteolytic processing

Transcription and translation reactions were performed similar to those described previously using a TnT T7 Coupled Reticulocyte Lysate System (Promega) except when noted below [13]. The reactions (25 µL) were carried out with Easy-Tag L-^35^S-methionine (>1000Ci/mM, Perkin Elmer) rather than ^35^S-cysteine. This was done since there are a similar number of methionines in the two products from the first cut and therefore they would yield bands with similar intensity in the autoradiograms. In addition, mutating the cysteines of the encoded protease would not alter the intensity of the product bands in experiments using these constructs. A master mix was prepared using the TnT rabbit reticulocyte lysate (RRL), reaction buffer, T7 polymerase, and amino acid mixture (minus methionine) provided with the system at ratios suggested by the technical bulletin. Additionally, RNasin Ribonuclease Inhibitor (Promega) was added to the master mix at 1 µL per 25 µL reaction mixture. Nuclease-free DEPC water was used to bring the final volume of each reaction to 25 µL. The master mix was incubated on a heat block for 15 minute at 30°C. The DNA plasmid constructs were added to the master mix at 0.5 µg/reaction or at 0.125 µg/reaction for pMA/CA/p2. When multiple plasmids were tested in the same experiment, the master mix was distributed among the treatment wells before the addition of DNA. Purified HIV-1 protease was prepared as described previously [35] and when indicated added at a final concentration of 250 nM active protease dimer. After incubation at 30°C, an equal volume of 3x lithium dodecyl sulfate-polyacrylamide gel electrophoresis (LDS-PAGE) loading buffer containing 150 mM dithiothreitol (DTT) (Invitrogen) was added to each reaction tube and the samples were heated at 70°C for 15 minutes. The samples were cooled at room temperature for 30 minutes and then loaded onto a 1 mm 10-well 4–12% Bis-Tris NuPAGE gel (Invitrogen). The gel was run at 100 volts for the first 10 minutes and then run at 200 volts for 70 minutes. The gel was washed in the following sequence; distilled water for 10 minutes, 10% acetic acid for 30 minutes, 8–10 washes with distilled water, and finally 10% glycerol for 15 minutes. Subsequently, the gel was placed under a conventional dryer/vacuum for 90 minutes at 54°C and exposed to film for autoradiography using Kodak Scientific Imaging film (Kodak). For quantification, GagProPol related bands on the film were scanned and densitometry performed using Un-Scan-It (Silk Scientific Corporation). Ratios of product formation to total GagProPol protein production (substrates and products) for each sample were determined, thus correcting for any internal differences in the overall extent of translated GagProPol protein that was obtained for each sample.

## Supporting Information

Figure S1 In vitro transcription/translation with 35S-methionine was carried out for 45, 60, 75 and 90 minutes without (lanes 1,3,5,and 7) or with (lanes 2,4,6, and 8) 1 mM DTT. Samples were separated by LDS-PAGE and visualized by autoradiography and the percent processing determined using densitometry. The precursors (GagProPol and GagProPolii) and two products (MA-p2, NC-IN) are indicated.
(2.18 MB TIF)
