# Supplementary figures and images for: The Initial Step in Human Immunodeficiency Virus Type 1 GagProPol Processing Can Be Regulated by Reversible Oxidation
Source: PLoS One. 2010 Oct 22;5(10):e13595. doi: 10.1371/journal.pone.0013595 (PMC2962637; doi:10.1371/journal.pone.0013595)

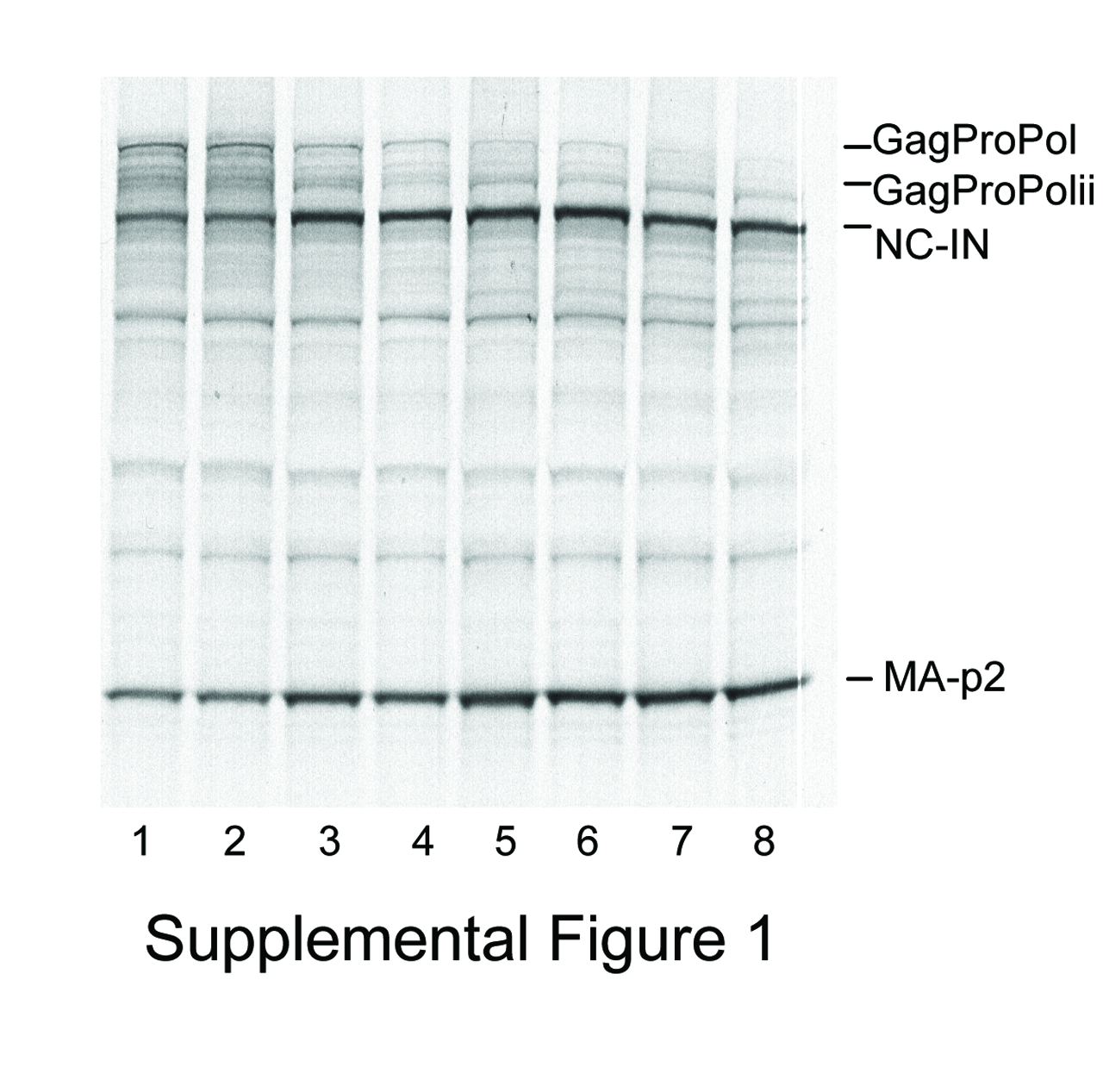

Supplement: Figure S1 — In vitro transcription/translation with 35S-methionine was carried out for 45, 60, 75 and 90 minutes without (lanes 1,3,5,and 7) or with (lanes 2,4,6, and 8) 1 mM DTT. Samples were separated by LDS-PAGE and visualized by autoradiography and the percent processing determined using densitometry. The precursors (GagProPol and GagProPolii) and two products (MA-p2, NC-IN) are indicated. (2.18 MB TIF) [file pone.0013595.s001.tif]
